# Evaluation of In Vitro and In Vivo Pharmacological Activity of *Elatostema sessile* With In Silico Approaches

**DOI:** 10.1002/fsn3.70052

**Published:** 2025-03-06

**Authors:** Aninda Kumar Nath, Saima Sultana Alam, Joushan Ara, Md. Mustafiz Chowdhury, Ummah Tasnim Nisat, Mohammad Jamal Uddin, Md. Tanvir Chowdhury, Sabbir Khan, Mycal Dutta

**Affiliations:** ^1^ Department of Pharmacy University of Science and Technology (USTC) Chittagong Bangladesh; ^2^ Department of Pharmacy, Faculty of Biological Sciences University of Chittagong Chittagong Bangladesh; ^3^ Department of Botany, Faculty of Biological Sciences University of Chittagong Chittagong Bangladesh

**Keywords:** analgesic, CNS depressant, *Elatostema sessile*, GC–MS, herbal medicine, molecular docking

## Abstract

*Elatostema sessile* is used by traditional medicinal practitioners to treat various diseases. The methanol extract (MES) and its fractions, including petroleum ether (PES), carbon tetrachloride (TES), chloroform (CES), and aqueous soluble fractions (AES), were tested for their antioxidant, cytotoxic, analgesic, and sedative properties on Swiss albino mice. In addition, we used GCMS to determine the bioactive compounds present in the MES of *E. sessile* . The MES had the lowest IC_50_ value (24.95 μg/mL) for antioxidant activity, measured by DPPH free radical scavenging, whereas the IC_50_ value for ascorbic acid was 5.80 μg/mL. In cytotoxic test with brine shrimp, PES exhibited the lowest LC_50_ value of 6.10 μg/mL among all the extract. Using the formalin‐induced paw licking method, MES and PES showed substantial results (*p* < 0.001) at 400 mg/kg dose. The tail immersion test showed significant (*p* < 0.05) findings for MES, PES, and TES after 30, 60, and 90 min following 400 mg/kg dose administration. The hole‐cross test demonstrated highly statistically significant results (*p* < 0.001) for the sedative activity of PES at a dose of 400 mg/kg after 30 min of administration and for CES after 90 min. According to molecular docking investigations, the extract has the potential to function as a pain‐relieving drug by inhibiting the enzymatic activity of cyclooxygenases 1 and 2. In summary, the results indicate that *E. sessile* offers significant therapeutic promise in the creation of innovative therapies for cancer, pain, and sleep problems.

## Graphical abstract

*Elatostema sessile* exhibits remarkable antioxidant properties, effectively scavenging free radicals and reducing oxidative stress, crucial in preventing various diseases. Additionally, its cytotoxic potential suggests promising anticancer applications, while its analgesic effects help in pain management by modulating nociceptive pathways. Furthermore, the plant demonstrates sedative activity, contributing to CNS depression and relaxation, making it a valuable candidate for developing natural therapeutic agents in pharmacology and traditional medicine.

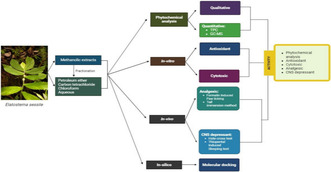

**Abbreviations**
AES: aqueous fractions of *E. sessile*

CES: chloroform fractions of *E. sessile*

CNS: central nervous system
COX: cyclooxygenase
DPPH: 2, 2‐diphenyl‐1‐picryl‐hydrazyl
GA: gallic acid
GABA: gamma‐aminobutyric acid
LD_50_
: lethal dose
MES: methanolic extract of *E. sessile*

MPE: maximum possible effect
PES: petroleum ether fraction of *E. sessile*

PRT: pain response time
ROS: reactive oxygen species
SOD: superoxide dismutase
TES: carbon tetrachloride fractions of *E. sessile*

TPC: total phenolic content

## 1 Introduction

Throughout history, people have used plants, animals, microbes, and aquatic species to cure illnesses. Humans used plants for medicinal purposes at least 60,000 years ago, according to the fossil data (Yuan et al. 2016). Researchers are investigating methods to treat, cure, mitigate, and prevent medical conditions (Patwardhan 2010). Synthetic and semi‐synthetic drugs have the potential to cause fatalities and result in hospitalization. Adverse medication reactions account for 8% of hospital admissions in the United States (Rupak et al. 2022). Researchers are now investigating plant‐based herbal medicines and formulating herbal medications to treat pain, diarrhea, diabetes, and several other ailments. Plants include several chemical compounds such as phenols, alkaloids, glycosides, saponins, tannins, terpenoids, polysaccharides, flavonoids, plant lipids, resins, and essential oils (Yang and Stöckigt 2010; Saxena et al. 2013). Oxidative stress is defined by elevated levels of reactive oxygen species (ROS) and insufficient antioxidant mechanisms. Cellular damage and tissue death may occur as a result of reactive stress (Preiser 2012). Researchers have extensively studied SOD, epigallocatechin‐3‐O‐gallate, CAT, lycopene, coenzyme Q10, ellagic acid, quercetin, indole‐3‐carbinol, genistein, vitamin E, and vitamin C as prophylactic and therapeutic drugs (Ratnam et al. 2006). Cancer is a lethal disease characterized by abnormal cellular behavior, unregulated cell division and proliferation, and ultimately, cell death (Uddin et al. 2020). Presently, traditional therapeutic methods, including surgical intervention, radiation therapy, and chemotherapy, all possess certain limitations. Natural cytotoxic chemicals have the potential to aid in cancer therapy by selectively damaging cancer cells, which is encouraging. Damage to tissue can cause an unpleasant sensation known as pain or algesia. Pain, although initially functioning as a safeguard, may eventually do more harm than good (Schug et al. 2018). Analgesics alleviate pain by exerting their effects on either the peripheral or central nervous systems (Sen et al. 2010). Nonsteroidal anti‐inflammatory medications (NSAIDs) and opioids are two types of traditional pain therapies (Hewitt et al. 2009). Anxiety and depression are common mental illnesses that often occur together and are consequently classified as internalizing disorders (Vink et al. 2008). Often, medication is used to treat anxiety disorders. Some often prescribed alternatives include SSRIs, SNRIs, pregabalin, buspirone, tricyclic antidepressants, monoamine oxidase inhibitors, and benzodiazepines (Bandelow et al. 2017). Although these artificially produced antidepressants and calming therapies have been shown to be effective, many patients discontinue their usage due to the occurrence of adverse effects or a substantial delay in their effectiveness. Throughout history, herbal treatments have been used for their therapeutic properties, serving as a fundamental component of traditional medicine (Pan et al. 2014). Approximately 500 medicinal plants are mentioned in ancient texts, whereas traditional medicine uses over 800 different plant species (Chopra 1956). Upon the introduction of conventional pharmaceuticals, scientists started to scrutinize the safety of traditional medicine, mostly owing to insufficient scientific evidence. During the 19th century, individuals endeavored to isolate the potent components of medicinal plants and succeeded in extracting quinine from Cinchona bark (Phillipson 2001). The convincing results prompted scientists to rely on this alternative type of treatment and undertake more investigations. Moreover, it is increasingly becoming popular among the general populace because of its superior effectiveness, fewer health hazards, and cheaper expenses compared to conventional therapy. As a result, phytochemistry has played a significant role in creating powerful conventional medicines extracted from plants. These include paclitaxel, vinblastine, and vincristine, which are used to treat cancer (Cragg and Newman 2005), as well as morphine, a narcotic painkiller (Rates 2001), and quinine and artemisinin, which are used to treat malaria (Queiroz et al. 2009).

*Elatostema sessile* is a genus of the rose plant. It is a member of both the *Elatostema* genus and the Urticaceae family (Hassler 2019). It is a gregarious plant that thrives on damp rocks in shaded areas, with its roots developing under the surface. The branchlets may reach a length of up to 40 cm and have a zig‐zag shape. The leaves are arranged in an alternating pattern and are obliquely lance shaped, measuring up to 10 × 3 cm. They have almost no stems. The base is obtuse, with a border that has more than eight deep teeth and an apex that is sharp. The stipules are lanceolate (Elatostema Sessile‐Stalkless Elatostema 2013). The evergreen forests of Assam, India, and Indo‐Malesia are home to the species (Elatostema sessile J.R. Forster & G. Forster). People use the leaves of this plant as a poultice to treat gastrointestinal illnesses and alleviate bodily discomfort. Additionally, a paste made from the plant is applied to boils, pimples, and blisters (Upadhyay et al. 2021). Previous studies on the Elatostema species have identified a diverse range of phytochemicals, including phenolic compounds, flavonoids (Reza et al. 2018), alkaloids, saponins, steroids, tannins, glycosides, and other substances (Uddin et al. 2021). The elatostema species has shown a range of pharmacological effects, such as antioxidant, cholinesterase, analgesic, CNS‐depressing, anti‐inflammatory, antibacterial, and thrombolytic activity (Reza et al. 2018; Ali Reza et al. 2018; Uddin et al. 2019; Karim et al. 2015; Mariani et al. 2016). Unfortunately, our plant, *E. sessile*, did not undergo any phytochemical or pharmacological studies.

Therefore, the experiment was designed to reveal the concentration of phytochemicals as well as in vitro and in vivo pharmacological activity like antioxidant, cytotoxic, analgesic, and CNS‐depressant characteristics of crude and different fractions of *E. sessile* .

## 2 Methods

### 2.1 Chemicals

We used high‐quality chemicals such as gallic acid (GA), DPPH, methanol, petroleum ether, carbon tetrachloride, and chloroform to isolate and extract plant components for both in vitro and in vivo pharmacological investigations. Merck, a German‐based company, provided the compounds.

### 2.2 Collection and Identification of the Plant

A famous local traditional healer helped acquire the mature plant in August, 2023. It was recognized as *E. sessile* by a renowned taxonomist, who assigned the herbarium number KN‐100112.

### 2.3 Preparation of Crude Extracts

The plant components were cleaned, cut into smaller pieces, and left to desiccate in a shaded area for 8 days. The technique included immersing 700 g of pulverized *E. sessile* in 3.5 L of methanol. Stuart in the UK manufactured the filters and used a rotary evaporator at reduced pressure to concentrate the filtrate. The crude methanol extracts (MESs) were prepared using a total of 18.28 g of *E. sessile* whole plant.

### 2.4 Solvent–Solvent Partitioning

The MESs were divided into different fractions using a sequential solvent‐solvent partitioning method. The fractions were obtained by using petrolium ether, carbon tetrachloride, chloroform, and water, following the procedure developed by Kupchan et al. (1973) and a modified version of VanWagenen et al. (1993).

### 2.5 GC–MS Analysis

An Agilent Technologies mass spectrometer, located in Santa Clara, CA, USA, was used to examine the chemical components of the *E. sessile* extract. This analysis was conducted using 7890A capillary gas chromatography equipment. To conduct the analysis, ~6 μL of crude extract (1% weight/volume) was mixed with a solution of methanol, chloroform, and water in a ratio of 2.5:1:1. This mixture was then injected onto a fused silica capillary column (HP‐5MSI, 90 m × 0.25 mm) that had been coated with a thin film measuring 0.25 μm. The film was made up of 95% dimethylpolysiloxane and 5% phenyl. The carrier gas utilized was helium, with a purity of 99.999%. It was flowing at a rate of 1 mL/min. The mass chromatogram was examined using the MSquad at a temperature of 150°C, whereas the source temperatures were set at 250°C. The NIST mass spectrometry data center functions as a point of reference for identifying the chemical constituents of the extracts by comparing their MS spectra to the NIST database (Al‐Nuri et al. 2022).

### 2.6 In Silico Study

#### 2.6.1 Ligand Preparation

A selection of 10 prominent compounds was made from the GC–MS profiling for the purpose of conducting a molecular docking analysis to evaluate the effectiveness of the analgesic properties. These included Stigmasta‐5, 22‐dien‐3‐ol, acetate (3‐beta.); Retinoic acid; Lilial, 3‐beta‐Hydroxy‐5‐cholen‐24‐oic acid; Androstane‐3,17‐diol; 3‐beta, 5‐alpha,17‐beta, alpha‐Apocholic acid; dl‐Menthol; Levomenthol; Cyclopropaneoctanoic acid, 2‐hexyl, methyl ester; 9,12‐Octadecadienoic acid (Z,Z), methyl ester; and Alpha Linolenic Acid. All of the compounds were obtained in SDF format from the PubChem database. Then, using OpenBabel (version 2.3.1), the structures were exported to the pdb for‐mat. AutoDock Tools (version 1.5.6)'s ligand preparation module was used to convert these pdb files into pdbqt format.

#### 2.6.2 Protein Preparation

The three‐dimensional structures of cyclooxygenase‐1 (PDB ID: 5wbe) (Cingolani et al. 2017) and human cyclooxygenase‐2 (PDB ID: 5ikq) (Orlando and Malkowski 2016) were obtained in PDB format from the protein data bank (Berman et al. 2000) for the evaluation of analgesic action. Discovery Studio 2020 prepared the protein structures by removing water molecules and complicated co‐structures. The proteins were subjected to an energy reduction technique using the steepest descent and conjugate gradient methods. The GROMOS 96 43B1 parameters were used to perform energy calculations using the Swiss‐PDB Viewer (Version 4.1.0) in a vacuum. AutoDock Tools (version 1.5.6) converted the pdb format to the pdbqt format and saved the resulting macromolecules in this format.

#### 2.6.3 Molecular Docking Simulation

Molecular docking investigations were conducted using Autodock Vina (version 1.1.2) to ascertain the mechanism by which the chosen drugs inhibit analgesia via the blockade of COX‐1 and COX‐2 enzymes. The grid box center for cyclooxygenase‐1 were 37.2833 in the X direction, 163.0440 in the Y direction, and 27.4380 in the Z direction. For human cyclooxygenase‐2, the grid box center were 21.8666 in the X direction, 51.6500 in the Y direction, and 17.1796 in the Z direction. The shell script provided by the creators of AutoDock Vina was used to implement Autodock Vina, and the ligands' binding affinity was quantified in terms of kcal/mol (Kumar et al. 2023; Trott and Olson 2010).

#### 2.6.4 Qualitative Phytochemical Screening

The qualitative analysis revealed the presence of terpenoids, saponins, flavonoids, phenol, tannins, glycosides, phlobatannins, steroids, anthraquinones, alkaloids, resins, cardiac glycosides, carbohydrates, proteins, fat and oil, and coumarin, in accordance with established methodology (Shaikh and Patil 2020). Analytical responses to these qualitative assessments were determined by the intensity of color or the occurrence of precipitate formation.

#### 2.6.5 Determination of Total Phenolic Content

The total phenolic content (TPC) was determined using the spectrophotometric technique (Kim et al. 2003). A 1 mL solution containing a combination of Folin–Ciocalteu's phenol reagent and 1 mL of sample with a concentration of 1 mg/mL was used for summarization. After being incubated at room temperature for 5 min, the mixture was added to 10 mL of a 7% Na_2_CO_3_ solution, 13 mL of deionized distilled water, and then well mixed. After a period of 90 min in the complete absence of light at a temperature of 23°C, the mixture was examined to determine its absorbance at a wavelength of 750 nm. The TPC was determined by extrapolating the data obtained from a calibration curve constructed using a GA solution. The estimation of the phenolic compound was conducted on three separate occasions. The phenolic content was measured in milligrams of gallic acid equivalents (GAE) per gram of dry material.

#### 2.6.6 Free Radical Scavenging Activity (DPPH)

Blois' approach quantitatively assessed the radical scavenging capacities of *E. sessile* whole crude methanol extracts (Blois 1958. In summary, a 0.1 mM solution of 2, 2‐diphenyl‐1‐picryl‐hydrazyl (DPPH) in methanol was prepared. Then, 1 mL of this solution was combined with 3 mL of extracts at different concentrations ranging from 500 to 0.49 μg/mL. As a reference or standard, ascorbic acid was used. The discoloration was seen at a wavelength of 517 nm after being in darkness for 30 min. The measurements were acquired on three separate occasions. The equation provided was used to calculate the capacity to scavenge the DPPH free radical, which was then expressed as a percentage of inhibition.

$$\begin{array}{c} \%\text{Inhibition}=(\text{Absorbance of control}–\text{Absorbance of test}/\\ \;\text{Absorbance of control})\times 100 \end{array}$$

Using a regression equation developed from data collected at varying concentrations of methanol extracts, we were able to determine the IC_50_ values (the concentration of sample necessary to scavenge 50% of free radicals).

### 2.7 Evaluation of Cytotoxic Activity

#### 2.7.1 Preparation of Seawater

After dissolving 38 g of sea salt (iodine‐free) in 1 L of distilled water, the solution was filtered to remove impurities. Using a 1 N NaOH solution, we could keep the seawater's pH stable between 8.0 and 8.5 (Mosaddik and Haque 2003).

#### 2.7.2 Brine Shrimp Eggs Hatching

Pet shops provided *Artemia salina* leach, commonly known as brine shrimp eggs, for the experiment. The little aquarium was filled with saline water and then populated with prawn eggs. The nauplii would hatch from the eggs after a period of 2 days. During the hatching process, the oxygen pump maintained a consistent and continuous supply. The prawn nauplii were taken out from the illuminated section of the tank due to their attraction to light, a behavior known as phototaxis. The nauplii were extracted from the tank using a pipette. In this study, 10 nauplii were put into 5 mL of saltwater and then exposed to plant extract solutions with 1000, 800, 500, 250, 125, 62.5, and 31.25 μg/mL concentrations. After a duration of 24 h, the Petri plates were inspected using a black light and a magnifying lens to count the quantity of surviving nauplii. In this bioassay, death was operationally defined as the absence of synchronized forward locomotion for a duration of 30 s. The equation (Sathasivam and Lakshmi 2017) was used to compute the mortality percentage (%) of brine shrimp nauplii at each concentration.

$$\%\text{Mortality}=N_{d}/N\times 100$$

Here, *N*_d_ is the number of dead nauplii and *N* is the number of nauplii taken.

#### 2.7.3 Determination of Median Lethal Concentration

The median lethal concentration (LC_50_) value was determined by using a linear regression model to predict the concentration of the extract at which 50% of brine shrimp nauplii perished after exposure to the drug for a certain duration. A graph revealed a linear relationship between the concentration and the percentage of mortality. A line of trend‐fitted linear regression analysis converted the concentration‐response data into a linear format with using Microsoft Excel 2007. The LC_50_ results were determined by extending the line of greatest fit.

#### 2.7.4 Acute Toxicity Study

Following the previously described methods, a study was conducted to investigate acute toxicity (Al‐Araby et al. 2020). After fasting overnight, each set of five Swiss albino mice received extracts. Various groups of animals were given oral doses of 1000, 2000, 3000, and 4000 mg/kg of body weight for each extract. Following the administration of the plant extract, a period of 3–4 h was observed during which eating was prohibited. Following the first 30 min, the animals were observed for the initial 24 h and then for the subsequent 3 days. On a daily basis, the mice were thoroughly examined for any alterations in their skin, hair, mucous membranes, eyes, respiration rate, heart rate, central nervous system, and autonomic nervous system. The effective dose would be a fraction of the LD_50_.

#### 2.7.5 Experimental Animals

To ascertain the analgesic and sedative effects, a total of 240 animals were first weighed, then re‐weighed, and subsequently divided into 12 distinct subgroups. The treatment conditions consisted of a saline control group (Group I), a group receiving conventional medicine (Group II), and other groups that were given different doses (200 and 400 mg/kg) of MES and various fractions. The research participants consisted of male Swiss Albino mice, with an average weight ranging from 20 to 30 g. Their acclimation phase lasted 14 days in standard propylene cages under controlled conditions (room temperature of 25°C ± 2°C, relative humidity of 60%–70%). Subsequently, they were exposed to a 12‐h alternation between light and darkness while being provided with pellets for nourishment. The study provided the mice with nourishing food and unrestricted access to water throughout. After each session, all of the mice were euthanized using chloroform anesthesia. This report adheres rigorously to the ARRIVE Guidelines for reporting research using animals. With authorization number USTC/AEC/24/010 the ethical committee has thoroughly examined and approved all experiments.

#### 2.7.6 Experimental Design for In Vivo Testing

A total of 12 groups of mice were used for the analgesic activity test, with five mice selected for each group. Group (I) was administered 10 mL/kg of a 1% tween−80 solution as a control. Group II was administered 40 mg/kg of ibuprofen for the formalin‐induced paw licking test and 10 mg/kg of morphine sulphate for the tail immersion. Similarly, other groups were given 200 and 400 mg/kg of crude MES and its different fractions. A total of 60 experimental mice were selected, assigned numbers, measured for weight, and then divided into 12 groups to study sedative effects. Group I was designated as the control group and administered saline. Group II was given the conventional medication diazepam at a dose of 1.0 mg/kg. The remaining groups received varying amounts (200 and 400 mg/kg) of methanol extract derived from *E. sessile* , together with petroleum ether (PES), carbon tetrachloride (TES), chloroform (CES), and aqueous soluble fractions (AES). After each trial, the mice that had received treatment were euthanized using diethyl ether anesthesia.

### 2.8 Evaluation of Analgesic Activity

#### 2.8.1 Formalin Induced Paw Licking in Mice

*Elatostema sessile* was tested for its ability to elicit inflammatory pain using a formalin‐induced pain paradigm in mice (Buisson and Dennis 1977), with some adjustments made. A total of 60 mice were randomly allocated into twelve groups, with each group consisting of 5 mice. After a 1‐h oral pretreatment, mice were administered either a vehicle *E. sessile* at doses of 200 and 400 mg/kg or ibuprofen (at a dose of 40 mg/kg) by intraperitoneal injection. Following this, a 0.05‐mL dose of commercially available 37% formalin was administered on the dorsal surface of the left hind paw in rats. The duration of licking was measured over a period of 30 min after the introduction of formalin. The first phase, occurring at 10 min, reflects the neurogenic pain response, whereas the latter phases at 10 and 30 min indicate the inflammatory pain response. The analgesic action was determined by calculating the percentage inhibition at both the early and late phases using the following formula:

$$\text{Percentage inhibition}=\frac{\mathrm{Nc}-\mathrm{Nt}}{\mathrm{Nc}}\times 100$$

where Nc is average licking (s) in control and Nt is the average licking (s) in test.

#### 2.8.2 Tail Immersion Method

Using a thermal methodology, we successfully evaluated the analgesic effects of the researched extracts on the central nervous system (Di Stasi et al. 1988). Before the 30‐min treatment period, we measured the duration it took for each mouse to withdraw its tail from water that was heated to a temperature of 50°C ± 1°C. Each mouse's tail was submerged for about two to three centimeters. The animals chosen for the investigation were those capable of promptly exhibiting a flicking motion within a timeframe of 3–5 s. A timer was programmed for a duration of 15 s to deactivate the tail protection mechanism. The evaluation of medicated mice was done at 30, 60, 90, and 120 min after the initiation of treatment (Malairajan et al. 2006). To get measurements, the animals were temporarily immobilized by gently wrapping them. At the end of each observation period, all mice that had received treatment were euthanized using diethyl ether anesthesia. The equation (Fan et al. 2014) was used to determine the analgesic effectiveness of the extract, expressed as a percentage of the maximum possible effect (% MPE).

$$\begin{array}{c} \mathrm{MPE}\;\left(\%\right)=\left(\text{Post drug latency}-\mathrm{Pre}\;\text{drug latency}\right)/\\ \;\left(\mathrm{Cut}\;\mathrm{off}\;\text{time}-\mathrm{Pre}\;\text{drug latency}\right)\times 100 \end{array}$$

The percentage of time elongation was calculated from the following equation:

$$\begin{array}{c} \text{Elongation}\;\left(\%\right)=\left(\text{Latency of Test}-\text{Latency of control}\right)/\\ \;\text{Latency of test}\times 100 \end{array}$$

### 2.9 Sedative Activity

#### 2.9.1 Hole‐Cross Test Apparatus

The experiment was conducted inside a square wooden box measuring 30 × 20 × 14 cm, which was devoid of a roof (Takagi et al. 1971). A robust wooden barrier divides the area in half at the center. A round aperture of 3.5 cm in diameter and 7.5 cm in height was included in the immobile wooden barricade. We used a tally counter to document the frequency of mice traversing between the distinct compartments at regular intervals of 0, 30, 90, and 120 min.

#### 2.9.2 Thiopental Sodium‐Induced Sleeping Test

After a period of 30 min, all mice were administered thiopental sodium (40 mg/kg) to induce sleep after the application of treatments. The time delay between the delivery of thiopental and the occurrence of the loss of the corrective reflex, sleep start, and sleep length was documented (Al Mahmud et al. 2016).

#### 2.9.3 Statistical Analysis

The data were shown as the mean ± SEM (standard error of the mean). The statistical analysis was conducted using the Statistical Package for the Social Sciences (SPSS, Version 16.0, IBM Corporation, New York). A one‐way ANOVA was done, followed by a post hoc Dunnett's test for comparisons. The significance thresholds were determined as follows: *p < 0.05, **p < 0.01, and ***p < 0.001. These values indicate statistical significance when compared to the control group.

## 3 Results

### 3.1 Qualitative Phytochemical Screening

This study examined the extracts to see if they included any initial bioactive phytochemicals. The findings, shown in Table 1, indicate that the extracts contained various phytochemicals. Table 1 displays the comprehensive results of the phytochemical examinations conducted on various components of intact *E. sessile* plants.

**TABLE 1:** Preliminary screening of bioactive phytochemicals of the examined extracts of *E. sessile* whole plant.

Phytochemicals	Methods	Crude (MES)	Petroleum ether (PES)	Carbon tetrachloride (TES)	Chloroform (CES)	Aqueous (AES)
Terpenoids	Salkowski's test	−	+	++	+++	+
	Liebermann–Burchard's test	+	+	++	+	+
Flavonoids	Alkaline reagent test	+	_	+	_	+
	Zinc hydrochloric acid reduction test	−	+	+	+	++
	Lead acetate solution test	+	++	++	+	−
	Sulfuric acid test	−	+	+	+	−
Saponins	Foam test	+	++	+	−	+
Phenols and tannins	Ferric chloride test	−	+	+++	+	+
Phlobatannins	Hydrochloric acid test	−	++	+	+	−
Steroid	Salkowski's test	−	++	+	+	−
	Liebermann‐Burchard's test	++	−	+	−	+
Anthraquinones	Acid test	−	+	+	++	−
	Hydroxy‐anthraquinone test	−	+++	−	++	−
Alkaloids	Mayer's test	+	+	+++	_	+
	Wagner's test	−	+	+	+	−
	Hager's test	−	+	+	−	+
Glycosides	Sodium hydroxide reagent test	+	−	−	−	−
	Liebermann's test	−	++	−	++++	+
	Salkowski's test	+	−	−	−	+
Cardiac glycosides	Keller‐kiliani test	−	+	−	+++	+
	Baljet's test	+	+	−	+	+
Resins	Acetone test	+	++	+++	+	−
Carbohydrates	Benedict's test	−	−	−	−	−
	Molisch's test	−	++	++	+	−
	Iodin test	++	−	−	−	++
	Fehling's test	+	−	+	−	+
Proteins	Biuret test Nitric acid test	+++	+++	+	+	+
Fats and fixed oils	Copper sulfate test	−	−	+	+	−
Coumarin	Sodium hydroxide test	+	−	−	++	−

*Note:* ‘+’ represents the presence of phytochemicals, and ‘−’ represents absence of phytochemicals.

### 3.2 GC–MS Profiling

**FIGURE 1:**
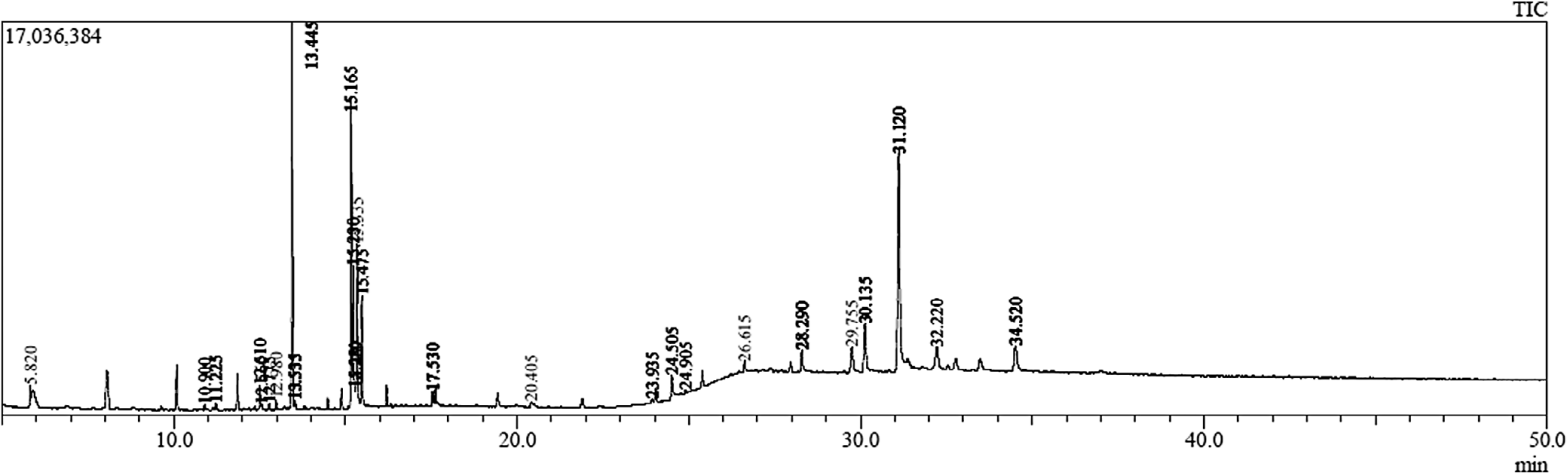
GC–MS chromatogram of methanol extract of *E. sessile*.

The methanolic extract of *E. sessile* , which is rich in phytoconstituents, was analyzed using gas chromatography–mass spectrometry (GC–MS). Forty molecules were discovered in the methanol extract, demonstrating a range of phytochemical activities. Figure 1 displays the chromatogram, whereas Table 2 provide a list of the chemical components together with their molecular formula, molecular weight (MW), retention time (RT), and area.

**TABLE 2:** Compounds identified by GC–MS analysis of methanol extract of *E. sessile*.

Sl. no.	Compound name	Molecular formula	Molecular weight (g/mol)	RT (min)	Area
1	2‐Propenoic acid, 2‐[(trimethylsilyl)oxy]‐3‐[4‐[(	C_18_H_32_O_4_Si_3_	396.7	5.822	413,102
2	2‐Nonyn‐1‐ol	C_9_H_16_O	140.22	10.603	38,181
3	2,7‐Octadiene‐1,6‐diol, 2,6‐dimethyl—	C_10_H_18_O_2_	170.25	10.603	38,181
4	Tetradecanoic acid, 12‐methyl‐, methyl ester	C_16_H_32_O_2_	256.42	11.225	145,702
5	Hexadecanoic acid, methyl ester	C_17_H_34_O_2_	270.5	11.225	145,702
6	Heneicosanoic acid, methyl ester	C_22_H_44_O_2_	340.6	11.225	145,702
7	Decanoic acid, methyl ester	C_11_H_22_O_2_	186.29	11.225	145,702
8	Docosanoic acid, methyl ester	C_23_H_46_O_2_	354.6	11.225	145,702
9	Eicosanoic acid, methyl ester	C_21_H_42_O_2_	326.6	11.225	145,702
10	Heptacosanoic acid, methyl ester	C_28_H_56_O_2_	424.7	11.225	145,702
11	Dodecanal	C_12_H_24_O	184.32	12.511	149,973
12	Undecanal	C_11_H_22_O	170.29	12.511	149,973
13	Phytol	C_20_H_40_O	296.5	12.775	20,315
14	*n*‐Nonadecanol‐1	C_19_H_40_O	284.5	12.775	20,315
15	Levomenthol	C_10_H_20_O	156.26	12.775	20,315
16	dl‐Menthol	C_10_H_20_O	156.26	12.775	20,315
17	cis‐9‐Tetradecen‐1‐ol	C_14_H_28_O	212.37	13.447	1,036,801
18	Hexadecanoic acid, methyl ester	C_17_H_34_O_2_	270.5	13.448	6,102,296
19	Heptadecanoic acid, methyl ester	C_18_H_36_O_2_	284.5	13.448	6,102,296
20	Pentadecanoic acid, methyl ester	C_16_H_32_O_2_	256.42	13.448	6,102,296
21	Dodecanoic acid, methyl ester	C_13_H_26_O_2_	214.34	13.448	6,102,296
22	Nonadecanoic acid, methyl ester	C_20_H_40_O_2_	312.5	13.448	6,102,296
23	Lilial	C_14_H_20_O	204.31	13.446	80,698
24	3.beta.‐Hydroxy‐5‐cholen‐24‐oic acid	C_24_H_38_O_3_	374.6	13.446	80,698
25	Stigmasta‐5,22‐dien‐3‐ol, acetate, (3.beta.)—	C_31_H_50_O_2_	454.7	13.446	80,698
26	.alpha.‐Apocholic acid	C_24_H_38_O_4_	390.6	13.446	80,698
27	Retinoic acid	C_20_H_28_O_2_	300.4	13.446	80,698
28	.beta. Carotene	C_40_H_56_	536.9	13.446	80,698
29	Androstane‐3,17‐diol, (3.beta.,5.alpha.,17.beta.)—	C_19_H_32_O_2_	292.5	13.446	80,698
30	L‐Alanine, N‐[(1,1‐dimethylethoxy)carbonyl]—	C_8_H_15_NO_4_	189.21	13.446	80,698
31	9,12‐Octadecadienoic acid (Z,Z)‐, methyl ester	C_19_H_34_O_2_	294.5	15.17	2,377,878
32	Linoleic acid ethyl ester	C_20_H_36_O_2_	308.5	15.17	2,377,878
33	12,15‐Octadecadienoic acid, methyl ester	C_19_H_34_O_2_	294.5	15.17	2,377,878
34	7‐Hexadecenoic acid, methyl ester, (Z)—	C_17_H_32_O_2_	268.4	15.169	1,390,508
35	6‐Octadecenoic acid, methyl ester, (Z)—	C_19_H_36_O_2_	296.5	15.169	1,390,508
36	9‐Octadecenoic acid, methyl ester, (E)—	C_19_H_36_O_2_	296.5	15.169	1,390,508
37	15‐Tetracosenoic acid, methyl ester, (Z)—	C_25_H_48_O_2_	380.6	15.169	1,390,508
38	9‐Octadecenoic acid (Z)‐, methyl ester	C_19_H_36_O_2_	296.5	15.169	1,390,508
39	11‐Octadecenoic acid, methyl ester	C_19_H_36_O_2_	296.5	15.169	1,390,508
40	Cyclopropaneoctanoic acid, 2‐hexyl‐, methyl ester	C_18_H_34_O_2_	282.5	15.169	1,390,508

### 3.3 Acute Toxicity Study

None of the investigated extracts induced any abnormal behaviors in the mice at the tested doses, such as reduced motor activity, seizures, agitation, unconsciousness, excessive tearing, or diarrhea. In the testing settings, no mice perished. Thus, it was concluded that the LD_50_ exceeded 4000 mg/kg.

### 3.4 Total Phenolic Content

Methanolic extract of *E. sessile* showed highest amount of phenolic compounds which is 73.00 ± 1.38 mg GAE/g and other fractions phenolic contents are shown in Table 3.

**TABLE 3:** Total phenolic content of crude and different fractions of *E. sessile*.

Group	Total phenol mg/g of extracts (GAE)
MES	73.00 ± 1.38
PES	49.58 ± 1.65
TES	60.44 ± 1.05
CES	72.00 ± 2.41
AES	67.25 ± 1.90

Abbreviations: AES, aqueous fractions of *E. sessile*, CES, chloroform fractions of *E. sessile* , MES, methanolic extract of *E. sessile*, PES, petroleum ether fraction of *E. sessile* , TES, carbon tetrachloride fractions of *E. sessile* .

### 3.5 Antioxidant Activity

To assess the efficacy of *E. sessile* crude and various fractions, we used a DPPH free radical scavenging experiment. The IC_50_ values, which quantify the concentration required to eliminate 50% of the DPPH free radicals, were used to assess the reduction capability of the components found in *E. sessile* . Table 4 displays the mean percentage of DPPH free‐radical scavenging activity at various doses. The MES had the lowest IC_50_ value of 24.95 μg/mL, whereas the standard ascorbic acid had an IC_50_ value of 5.80 μg/mL. The CES fraction has an IC_50_ value that is about equal to MES, namely 25.73 μg/mL.

**TABLE 4:** IC_50_ values of crude, different solvent fractions of *E. sessile* and ascorbic acid at different concentrations.

Sample	Free radical scavenging activity (IC_50_ μg/ml)
Ascorbic acid	5.80
MES	24.95
PES	52.88
TES	83.44
CES	25.73
AES	48.94

Abbreviations: AES, aqueous fractions of *E. sessile* , CES, chloroform fractions of *E. sessile* , MES, methanolic extract of *E. sessile*, PES, petroleum ether fraction of *E. sessile* , TES, carbon tetrachloride fractions of *E. sessile*.

### 3.6 Cytotoxic Activity

The brine shrimp test is used to quantify the cytotoxicity of a chemical. The LC_50_ of each sample was obtained after 24 h by graphing the percentage of surviving prawns against the logarithm of the sample concentration. The regression analysis was then used to get the best‐fit line from the curve data (Table 5). The standard medication vincristine sulfate had the lowest LC_50_ value of 0.45 μg/mL. The LC_50_ of PES is 6.10 μg/mL, which is the lowest among the other fractions. The LC_50_ values for MES, TES, CES, and AES are 45.54, 11.33, 14.04, and 57.62 μg/mL, respectively.

**TABLE 5:** LC_50_ value of methanol extract and different fractions of *E. sessile*.

Sample	(LC_50_ μg/ml)
Vincristin sulfate	0.45
MES	45.54
PES	6.10
TES	11.33
CES	14.04
AES	57.62

Abbreviations: AES, aqueous fractions of *E. sessile* , CES, chloroform fractions of *E. sessile* , MES, methanolic extract of *E. sessile*, PES, petroleum ether fraction of *E. sessile* , TES, carbon tetrachloride fractions of *E. sessile* .

### 3.7 Formalin‐Induced Paw Licking

The results of the one‐way ANOVA indicated that the crude extract and its various fractions of *E. sessile* had a substantial impact on the licking behavior of mice caused by formalin. The measurements of MES, PES, TES, and CES yielded statistically significant findings (*p* < 0.05) for both the 200 and 400 mg/kg doses. However, AES did not provide any statistically significant results at any dosage. The administration of MES and PES at a dosage of 400 mg/kg demonstrated a very statistically significant outcome (*p* < 0.001) at thesame dose (Table 6). Ibuprofen administered at a dosage of 10 mg/kg had a comparable impact on both the early phase licking (*p* < 0.001) and the late phase licking (*p* < 0.01) when compared to the control group treated with a vehicle.

**TABLE 6:** Analgesic activity of *E. sessile* and its different fractions in formalin induced paw licking test.

Groups	Licking time(s) Mean ± SEM		% inhibition	
	Early phase (0–10 min)	Late phase (10–30 min)	Early phase (0–10 min)	Late phase (10–30 min)
Control	280.21 ± 0.01	200 ± 0.01	—	—
Standard (Ibuprofen 40 mg/kg)	83.33 ± 0.12***	32.1 ± 0.08***	70.26	61.1
MES‐200	110.36 ± 0.89*	122.1 ± 0.87	35.01	28.3
MES‐400	76.12 ± 0.45***	88.2 ± 0.17*	45.21	35.4
PES‐200	122.09 ± 0.34	140.1 ± 0.45	57.19	41.7
PES‐400	80.12 ± 0.12***	92.6 ± 0.56**	76.12	65.3
TES‐200	192.52 ± 0.23*	210.5 ± 0.34	31.29	21.8
TES‐400	61.01 ± 0.23**	76.2 ± 0.19**	86.34	61.7
CES‐200	101.67 ± 0.45	120.3 ± 0.45	64.33	49.2
CES‐400	50.12 ± 0.76**	59.34 ± 0.12*	80.09	71.2
AES‐200	275.11 ± 0.45	300.01 ± 0.45	1.82	2.1
AES‐400	311.12 ± 0.45	288.02 ± 0.89	20.12	6.01

*Note:* Each value represents the mean ± SEM (*n* = 5). One‐way ANOVA followed by Dunnett's *t* test.

Abbreviations: AES, aqueous fractions of *E. sessile* , CES, chloroform fractions of *E. sessile* , MES, methanolic extract of *E. sessile*, PES, petroleum ether fraction of *E. sessile* , TES, carbon tetrachloride fractions of *E. sessile* .

* *p* < 0.05 compared with control.

** *p* < 0.01.

*** *p* < 0.001.

### 3.8 Tail Immersion Method

The analysis revealed that the extracts administered at doses of 200 and 400 mg/kg body weight exhibited distinct variations in pain response time (PRT), percentage of maximum potential effect (%MPE), and percentage elongation of delay when compared to the control group. The variations observed were directly proportional to the dosage. At time intervals of 30, 60, and 90 min after administering a dosage of 400 mg/kg, the outcomes of MES, PES, and TES showed statistically significant results (*p* < 0.05). However, CES and AES did not indicate any significant outcomes at any dosage (Table 7). Throughout the investigation, the proportion of MPE in these extracts remained consistently high. The level was significantly increased when given at the standard dosage of morphine sulfate (10 mg/kg). Table 8 displays the percentage increase in latency time. This evidence suggests that these extracts may have analgesic properties that affect both the peripheral and central nervous systems.

**TABLE 7:** Effects of methanol extracts and different fractions of *E. sessile* in tail immersion test in mice.

Test samples	Reaction times in seconds (mean ± SEM) and %MPE				
	Pre‐treatment	30 min	60 min	90 min	120 min
Control	2.13 ± 0.07 8.01%	2.50 ± 0.08 13.5%	2.13 ± 0.176 11.12%	2.7 ± 0.98 5.9%	2.9 ± 0.18 2.11%
Standard	2.36 ± 0.088 22.45%	4.8 ± 0.23 21.06%	8.20 ± 0.15* 24.06%	6.86 ± 0.16 10.22%	8.5 ± 0.10 8.01%
MES‐200	2.61 ± 0.059 4.14%	2.73 ± 0.20 2.06%	2.8 ± 0.2 4.22%	2.76 ± 0.12 3.77%	4.7 ± 0.10 2.84%
MES‐400	3.2 ± 0.49 12.15%	3.6 ± 0.09 15.12%	4.06 ± 0.79* 11.01%	4.77 ± 0.92 7.13*	4.6 ± 0.72* 5.46*
PES‐200	1.73 ± 0.14 8.88%	2.79 ± 0.067 5.8%	2.90 ± 0.88 3.9%	2.08 ± 0.36 4.8%	4.94 ± 0.14 2.21%
PES‐400	2.56 ± 0.13 12.45%	2.59 ± 0.80 10.12%	3.2 ± 0.35* 5.8%	4.0 ± 0.38 3.8%	8.9 ± 0.34 3.1%
TES‐200	2.77 ± 0.198 5.69%	2.5 ± 0.198 3.1%	5.1 ± 0.23 2.89%	2.64 ± 0.20 7.78%	2.19 ± 0.07 3.11%
TES‐400	8.6 ± 0.30*** 10.33%	7.86 ± 0.16 20.12%	2.53 ± 0.20 28.98%	4.7 ± 1.39 18.03%	2.6 ± 0.20 11.21%
CES‐200	4.6 ± 0.10 6.12%	2.76 ± 0.32 8.91%	2.3 ± 0.09 5.11%	2.09 ± 0.07 4.06%	2.1 ± 0.98 2.87%
CES‐400	7.1 ± 0.72 9.12	4.67 ± 0.2 14.7	5.59 ± 0.07 17.12	2.4 ± 0.90 10.09	7.16 ± 0.16 2.12%
AES‐200	2.79 ± 0.07 2.11%	2.0 ± 0.20 4.12%	2.7 ± 0.20 7.45%	2.3 ± 0.18 4.9%	6.2 ± 0.13 2.7%
AES‐400	4.3 ± 0.82 7.22%	4.77 ± 0.82 9.22%	3.89 ± 0.07 15.1%	2.1 ± 0.50 13%	6.17 ± 0.22 9.1%

*Note:* Each value represents the mean ± SEM (*n* = 5). One‐way ANOVA followed by Dunnett's *t* test.

Abbreviations: AES, aqueous fractions of *E. sessile* , CES, chloroform fractions of *E. sessile* , MES, methanolic extract of *E. sessile*, PES, petroleum ether fraction of *E. sessile* , TES, carbon tetrachloride fractions of *E. sessile* .

* *p* < 0.05 compared with control.

*** *p* < 0.001.

**TABLE 8:** Percent elongation of latency time after administration of all test samples.

Test samples	Dose (mg/kg)	% Elongation of latency time			
		30 min	60 min	90 min	120 min
Standard	50	51%	63.59%	64.9%	60.0%
MES‐200	200	7.77%	35.0%	16.10%	8.71%
MES‐400	400	21.09%	38.0%	41.4%	20.19%
PES‐200	200	32.2%	22.6%	32.12%	3.34%
PES‐400	400	25.3%	28.8%	41.8%	12.1%
TES‐200	200	2.04%	16.3%	32.2%	1.44%
TES‐400	400	28.62%	47.07%	25.03%	21.62%
CES‐200	200	3.44%	8.71%	2.94%	4.14%
CES‐400	400	23.6%	12.18%	21.62%	2.7%
AES‐200	200	12.08%	21.72%	3.34%	4.14%
AES‐400	400	20.2%	27.31%	4.45%	3.19%

Abbreviations: AES, aqueous fractions of *E. sessile* , CES, chloroform fractions of *E. sessile* , MES, methanolic extract of *E. sessile*, PES, petroleum ether fraction of *E. sessile* , TES, carbon tetrachloride fractions of *E. sessile* .

### 3.9 Hole Cross Test

The soluble fractions of MES, PES, TES, CES, and AES exhibited a statistically significant sedative effect (*p* < 0.05) compared to the other tested substances. Notably, the PES 400 mg/kg dose showed a significant effect at 30 min, and the CES 400 mg/kg dose at 90 min post‐administration, with a *p*‐value of less than 0.001. The results of hole cross test are presented in Table 9.

**TABLE 9:** Sedative activity by hole cross method of methanol extracts and different fractions of *E. sessile*.

Group	Number of movements			
	0 min	30 min	90 min	120 min
Control	8.5 ± 0.51	6.5 ± 0.31	4.7 ± 0.66	5.3 ± 0.18
Standard	16 ± 0.44***	5.1 ± 0.36	6 ± 0.71	8.2 ± 0.37
MES‐200	11 ± 0.71	7.2 ± 0.37	5.1 ± 0.51	10 ± 0.11
MES‐400	10 ± 0.12	4.8 ± 0.38**	5.8 ± 0.37	8.8 ± 0.16
PES‐200	16 ± 0.21	6.3 ± 0.17	5.5 ± 0.71	14 ± 0.11
PES‐400	9 ± 0.12	3.8 ± 0.87***	5.8 ± 0.57	12.8 ± 0.66
TES‐200	08 ± 0.11	10.1 ± 0.17	09 ± 0.21	16 ± 0.51
TES‐400	11 ± 0.72	4.5 ± 0.57	5.5 ± 0.77*	13.7 ± 0.66
CES‐200	13 ± 0.61	2.2 ± 0.77	3 ± 0.71	11 ± 0.51
CES‐400	7 ± 0.12	1.5 ± 0.97*	1.9 ± 0.17***	6.8 ± 0.16
AES‐200	14.8 ± 0.56	10.5 ± 0.97	11.8 ± 0.77	18 ± 0.7 3
AES‐400	12 ± 0.91	10.1 ± 0.57	18 ± 0.51*	20 ± 0.11

*Note:* Each value represents the mean ± SEM (*n* = 5). One‐way ANOVA followed by Dunnett's *t* test.

Abbreviations: AES, aqueous fractions of *E. sessile* , CES, chloroform fractions of *E. sessile* , MES, methanolic extract of *E. sessile*, PES, petroleum ether fraction of *E. sessile* , TES, carbon tetrachloride fractions of *E. sessile* .

* *p* < 0.05 compared with control.

** *p* < 0.01.

*** *p* < 0.001.

### 3.10 Thiopental‐Induced Sleeping Test

For the thiopental‐induced sleeping test, the study evaluated the time it took for sleep to begin (onset of sleep) and the length of sleep (duration of sleep) in subjects who were given the conventional medication diazepam and various components of *E. sessile*. The standard medicine had a very strong statistical effect (*p* < 0.001) on both the initiation and duration of sleep. The TES, CES, and AES demonstrated a high level of statistical significance (*p* < 0.001) as compared to the control group, namely in the case of the 400 mg/kg dosage (Table 10).

**TABLE 10:** Effects of treatment with the methanolic extract of *E. sessile* and different fractions in mice on thiopental sodium‐induced sleeping time.

Group	Onset of sleep (min)	Duration of sleep(min)
Control	18.82 ± 1.44	283.67 ± 1.19
Standard (Diazepam 1 mg/kg)	6.61 ± 0.70***	176.19 ± 2.4***
MES‐200	16.82 ± 0.54	250 ± 0.78
MES‐400	14.82 ± 0.44	224.47 ± 1.56
PES‐200	28.12 ± 0.94	240 ± 0.45
PES‐400	22.12 ± 0.54	207.42 ± 0.48
TES‐200	17.82 ± 0.94	197 ± 0.34
TES‐400	12.02 ± 0.78***	163.76 ± 2.69***
CES‐200	17.12 ± 0.74	199 ± 0.89*
CES‐400	10.02 ± 0.94***	157.81 ± 2.21***
AES‐200	20.12 ± 0.14	176 ± 0.90*
AES‐400	15.02 ± 0.14***	115.68 ± 1.06***

*Note:* Each value represents the mean ± SEM (*n* = 5). One‐way ANOVA followed by Dunnett's *t* test.

Abbreviations: MES, methanolic extract of *E. sessile*, PES, petroleum ether fraction of *E. sessile* , TES, carbon tetrachloride fractions of *E. sessile* , CES, chloroform fractions of *E. sessile* , AES, aqueous fractions of *E. sessile* .

* *p* < 0.05 compared with control.

*** *p* < 0.001.

### 3.11 Molecular Docking Analysis

**FIGURE 2:**
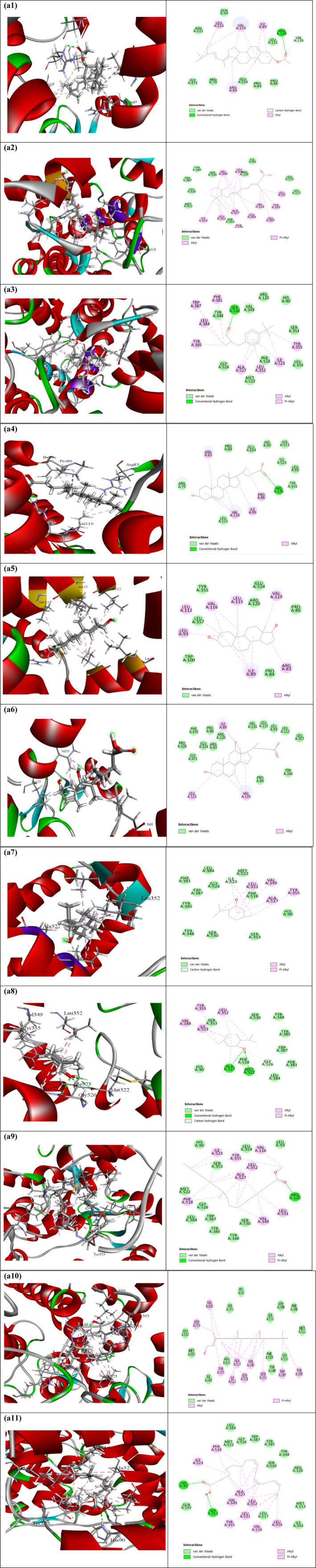
Molecular docking interaction of compounds against Cyclooxygenase‐1 (PDB ID: 5wbe): (a1) Stigmasta‐5,22‐dien‐3‐ol, acetate, (3.beta.)‐, (a2) Retinoic acid, (a3) Lilial, (a4) 3.beta.‐Hydroxy‐5‐cholen‐24‐oic acid, (a5) Androstane‐3,17‐diol, (3.beta.,5.alpha.,17.beta, (a6) .alpha.‐Apocholic acid, (a7) dl‐Menthol, (a8) Levomenthol, (a9) Cyclopropaneoctanoic acid, 2‐hexyl‐, methyl ester, (a10) 9,12‐Octadecadienoic acid (Z,Z)‐, methyl ester, and (a11) Alpha Linolenic Acid.

**FIGURE 3:**
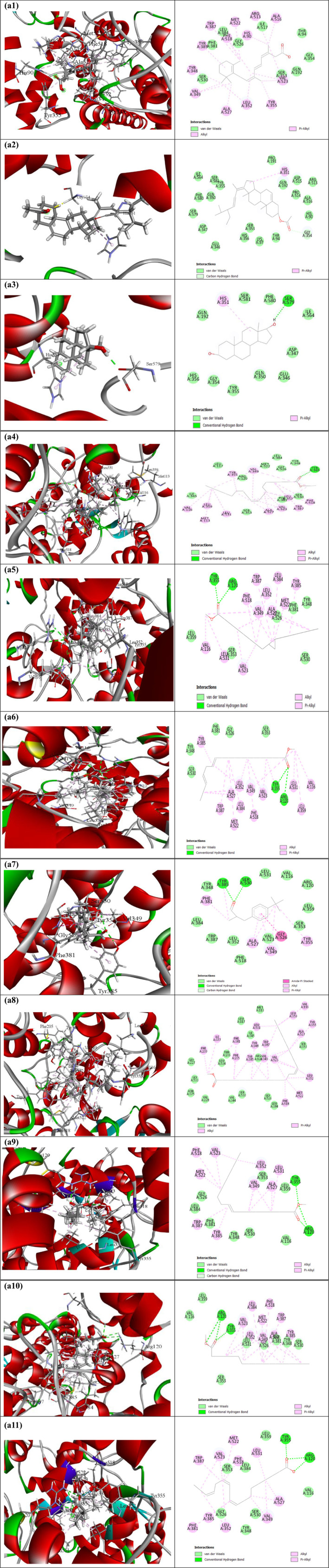
Molecular docking interaction of compounds against Cyclooxygenase‐2 (PDB ID: 5ikq): (a1) Retinoic acid, (a2) Stigmasta‐5,22‐dien‐3‐ol, acetate, (3.beta.)‐, (a3) Androstane‐3,17‐diol, (3.beta.,5.alpha.,17.betal, (a4) Linoleic acid ethyl ester, (a5) Cyclopropaneoctanoic acid, 2‐hexyl‐, methyl ester, (a6) 12,15‐Octadecadienoic acid, methyl ester, (a7) Lilial, (a8) 15‐Tetracosenoic acid, methyl ester, (Z)‐, (a9) 9‐Octadecenoic acid, methyl ester, (E)‐, (a10) Heptadecanoic acid, methyl ester, and (a11) Alpha‐linolenic acid.

Table 11, 12, along with Figures 2 and 3, presents the docking score and non‐bond interactions of the docked compounds for analgesic efficacy. Based on their docking scores, we chose 10 compounds to dock against the COX‐1 and COX‐2 receptors. Our molecular docking research revealed that each compound studied had interactions with all of the target proteins. As shown in Table 11, the COX‐1 enzyme (PDB ID: 5wbe) had the strongest binding power to Stigmasta‐5,22‐dien‐3‐ol, acetate, (3‐beta), and retinoic acid, with binding energies of −8 and −7.8 kcal/mol, respectively. In addition, several compounds exhibited a significant affinity for the COX‐1 receptor. All the compounds exhibited potent analgesic effects by inhibiting the COX‐2 receptor (PDB ID: 5ikq) in terms of COX‐2 inhibition. The substances Retinoic acid and Stigmasta‐5,22 dien‐3‐ol, acetate,(33‐beta) exhibited a high binding affinity of −7.4 (kcal/mol) as shown in Table 12. These findings show that *E. sessile* may exhibit potent analgesic action, as confirmed by an in vivo study.

**TABLE 11:** Binding affinity and interaction of the selected compounds with the amino acid residue of cyclooxygenase‐1 (PDB ID: 5wbe).

Compound name	Binding Affinity (kcal/mol)	Hydrogen bonds		Hydrophobic bonds	
		Conventional	Carbon‐hydrogen	Pi‐alkyl	Alkyl
Stigmasta‐5,22‐dien‐3‐ol, acetate, (3.beta.)—	−8	ARG120	VAL116	—	ILE89, VAL119 (2), ARG83 (2), ILE89, LEU123 (2), VAL119
Retinoic acid	−7.8	—	—	TYR355, PHE518	VAL349 (3), LEU352, ALA527 (4), VAL116 (2), LEU359, LEU531 (2), LEU352, VAL349, LEU531, LEU93, LEU357
Lilial	−7.4	SER530	—	TYR355, PHE381, TYR385, TRP387, LEU352, ILE523, ALA527	ALA527, LEU384, ILE523
3.beta.‐Hydroxy‐5‐cholen‐24‐oic acid	−7.2	ARG120 (2)	—	—	VAL119 (3), ILE89, ARG83, PRO86
Androstane‐3,17‐diol, (3.beta.,5.alpha.,17.beta	−7.2	—	—	—	ARG83, ILE89 (3), LEU115 (2), VAL116 (2), LEU93, LEU112, VAL119
.alpha.‐Apocholic acid	−6.9	—	—	—	ILE89 (2), VAL119 (3), LEU123
dl‐Menthol	−6.7	—	ILE523	TYR355	VAL349, LEU352 (2), ILE523 (2), ALA527
Levomenthol	−6.7	ALA527, MET522	GLY526	TYR355	VAL349, LEU352 (2), ILE523 (2), ALA527
Cyclopropaneoctanoic acid, 2‐hexyl‐, methyl ester	−6.7	ARG120	—	TYR355 (2), PHE518 (2)	VAL349 (2), ALA527 (3), LEU352 (3), ILE523 (2), LEU531, VAL116
9,12‐Octadecadienoic acid (Z,Z)‐, methyl ester	−6.4	—	—	TYR355 (2), TYR385, TRP387	VAL116 (2), VAL349 (3), ALA527 (3), ILE523, LEU352, LEU531 (2), LEU359
Alpha‐linolenic acid	−6.9	HIS90, SER353	—	TYR355, PHE518 (2)	VAL349 (3), ALA527 (3), LEU352 (3), ILE523, LEU531 (2), VAL116

**TABLE 12:** Binding affinity and interaction of the selected compounds with the amino acid residue of human cyclooxygenase‐2 (PDB ID: 5ikq).

Compounds	Binding Affinity (kcal/mol)	Hydrogen bonds		Hydrophobic bonds	
		Conventional	Carbon‐hydrogen	Pi‐alkyl	Alkyl
Retinoic acid	−7.4	—	—	HIS90, TYR348, TYR355, TYR385, TRP387, PHE518 (2)	LEU352 (2), ALA516, VAL523 (3), ALA527, LEU352, VAL349, MET522, ARG513
Stigmasta‐5,22‐dien‐3‐ol, acetate, (3‐beta.)—	−7.4	—	GLY354	HIS351	—
Androstane‐3,17‐diol, (3‐beta.,5‐alpha.,17‐beta	−7.3	SER579	—	HIS351	—
Linoleic acid ethyl ester	−7.1	TYR385	—	TYR355, TRP387, PHE518 (2)	VAL349 (3), VAL523 (2), ALA527 (3), LEU352 (3), MET113, VAL116, LEU359, LEU531
Cyclopropaneoctanoic acid, 2‐hexyl‐, methyl ester	−7	ARG120 (2), TYR355		TYR385, TRP387, PHE518 (2)	VAL523 (2), ALA527 (2), LEU352, LEU384, MET522, VAL349 (2), VAL116, LEU531
12,15‐Octadecadienoic acid, methyl ester	−6.9	ARG120 (2), TYR355	—	TYR355, TYR385, TRP387, PHE518	VAL523 (2), ALA527, LEU352 (2), VAL116, VAL349 (2), LEU359, LEU531, LEU384, MET522
Lilial	−6.9	TYR385, SER530	SER530	TYR355, PHE381, TYR385, VAL349, ALA527	ALA527, VAL349
15‐Tetracosenoic acid, methyl ester, (Z)—	−6.9	—	—	PHE205, PHE209, TYR348, TYR355, PHE381 (2), TYR385 (2), TRP387, PHE518	VAL349 (4), VAL523 (3), ALA527, ILE377, LEU352 (2), MET522, VAL116, LEU359, LEU531
9‐Octadecenoic acid, methyl ester, (E)—	−6.9	ARG120 (2), TYR355	ARG120	TYR385, TRP387, PHE518	VAL349 (2), VAL523 (2), ALA527 (2), LEU352 (2), LEU531, MET522
Heptadecanoic acid, methyl ester	−6.9	ARG120 (2), TYR355	—	TYR385 (2), TRP387, PHE518	VAL349 (2), VAL523 (2), ALA527 (2), LEU352 (3), LEU384, MET522
Alpha‐linolenic acid	−6.9	ARG120 (3), TYR355	—	PHE381, TYR385, TRP387, PHE518	VAL349 (2), VAL523 (2), ALA527 (2), LEU531, LEU352 (2), MET522

## 4 Discussion

People have long used herbal medicine to treat a wide range of ailments. This investigation evaluated the phytochemical investigation, GC–MS analysis, TPC, antioxidant activity, cytotoxicity, and in vivo analgesic and CNS depressing activities of the MES and its various fractions. During the qualitative phytochemical analysis test, the results revealed the presence of several phytochemical constituents in both the crude and fractionated forms. These constituents included alkaloids, carbohydrates, flavonoids, terpenoids, phenols, saponins, and other similar chemicals. Utilizing phytochemical screening procedures in the early stages may expedite the discovery of powerful phytochemicals, thereby aiding in the creation of innovative pharmaceutical therapies. Nowadays, people often view phenolic chemicals from plants as highly effective natural antioxidant sources. Different types of secondary metabolites are called phenolic compounds. They play a key role in protecting tissues from the harmful effects of free radicals, oxygen, and other reactive species. As a result, they hinder the development of several illnesses, including inflammatory disorders, cancer, diabetes, myocardial infarction, and Alzheimer's and Parkinson's diseases (Wachtel‐Galor 2011). A quantitative investigation of *E. sessile* MES identified phenolic components in both crude and crude fractions. The CES exhibited the highest levels of TPC, namely 73.00 ± 1.38 mg/g of extracts (GAE) and 72 ± 2.41 mg/g of extracts (GAE). Antioxidants, also known as free radical scavengers, protect cells in the body by interacting with and neutralizing free radicals, therefore preventing them from causing damage (Diplock et al. 1998).

The DPPH radical's activity is used to assess the capacity of various samples to scavenge free radicals. DPPH is a persistent hydrophilic free radical that appears violet and has its greatest absorbance at a wavelength of 515–517 nm. When DPPH accepts electrons from reducing chemicals like phenols, it undergoes a conversion into the compound hydrazine, which is colorless. A decrease in absorbance correlates with this structural alteration. Compounds with such potential are classified as antioxidants (Kim et al. 2019). In this work, MEP and CES showed potent antioxidant properties against DPPH free radicals. The IC_50_ values for the two substances were 24.95 and 25.73 μg/mL, respectively, whereas the IC_50_ value for ascorbic acid is 5.80 μg/mL. The research suggests that the high concentration of phenolic chemicals in some extracts, particularly polar extracts, may contribute to their increased antioxidant activity. There is compelling evidence indicating a direct relationship between the concentration of phenolic chemicals and the antioxidant activity of plants. Furthermore, it seems that phenolic chemicals, which are plentiful in plants with strong antioxidant properties, may primarily be obtained from plant extracts (Chotimarkorn et al. 2008). Recent evidence suggests that oxidative stress may play a role in the pathophysiology of various diseases, including inflammation, atherosclerosis, chronic renal failure, diabetes, and cancer (Kancheva and Kasaikina 2013). Several investigations have revealed the significance of phenolic compounds as scavengers of free radicals (Katalinic et al. 2006; Thériault et al. 2006). The polyphenolic components included in the extract may contribute to the process of scavenging free radicals.

The brine shrimp lethality test is a comprehensive method that assesses the cytotoxicity and diverse pharmacological effects of chemicals and plant preparations. These effects include antibacterial, pesticidal, antiviral, anticancer, and more (Meyer et al. 1982). The LC_50_ value for AES was 57.62 μg/mL, which is much higher than the reference drug's value of 0.45 μg/mL. This suggests that the plant extract is safe when used at therapeutic doses. Cell death was associated with secondary plant metabolites (Özçelik et al. 2011). An initial qualitative phytochemical analysis of *E. sessile* identified cancer‐fighting alkaloids, flavonoids, and tannins (Gordanian et al. 2014; Nobori et al. 1994). This association is appropriate for our current study.

The formalin test elicits a biphasic response. During the first 5 min after the formalin injection, the first phase, known as the early phase (neurogenic nociceptive response), takes place. The second phase, known as the inflammatory nociceptive response, often begins within 15 to 30 min after the injection of formalin. Research has shown that analgesic medications, such as opioids or centrally acting analgesics, have the ability to reduce pain‐related behaviors in both stages. Conversely, non‐steroidal anti‐inflammatory medications (NSAIDs) are only beneficial during the second phase (Pham and Spaniol 2024). In addition, the injection of formalin causes inflammation, characterized by the presence of redness and swelling in the injected paw during the inflammatory phase. The release of chemical mediators like histamine, bradykinin, serotonin, and substance causes this (Da Silva Prudêncio et al. 2025). The findings for MES, PES, TES, and CES were shown to be statistically significant (*p* < 0.05) for both the 200 and 400 mg/kg doses. However, AES did not provide any statistically significant results at any dosage. The administration of MES and PES at a dosage of 400 mg/kg demonstrated a very statistically significant outcome (*p* < 0.001) at the same dose of 400 mg/kg. Studies (Shajib et al. 2015; Ullah et al. 2014) have shown that the flavonoids, phenols, terpenoids, and polyphenols present in plants possess a soothing and pain‐relieving impact. Traditional chemotherapy approaches for treating cancer cells rely mostly on the features of medications to impair the capacity of cancer cells to constantly proliferate, reproduce, and eventually trigger cell damage and death.

Heat stimuli were administered during the assessment of the tail immersion model for central analgesic effect (Hada et al. 2024). According to this hypothesis, sensory nerves elevated pain thresholds (Shah and Shah 2015), made nociceptors more sensitive (Bachhav et al. 2009), and decreased prostaglandin participation. The current study found that higher levels of MPE, PRT, or latency time indicated the presence of analgesia from the drug or extract. Although the reference standard morphine sulfate (10 mg/kg) also increased those measures considerably, the doses of 200 and 400 mg/kg of MEA, PES, TES, CES, and AES showed a dose‐dependent increase in PRT, a higher percentage (%) of MPE, and elongation up to 120 min compared to the control. Due to the similarities in effects with morphine sulfate, it is plausible that the extracts functioned in a manner akin to opiates, perhaps alleviating pain via both spinal and supraspinal routes (Shah and Shah 2015). At time intervals of 30, 60, and 90 min after administration of a dosage of 400 mg/kg, the results of MES, PES, and TES were all found to be statistically significant (*p* < 0.05). However, CES and AES did not show any statistically significant outcomes at any dosage. This indicates that they have the potential to function in both the peripheral and central nervous systems. Animal studies have shown the pain‐relieving properties of polyphenolic compounds, such as flavonoids and tannins (Krasteva et al. 2007).

During the hole cross‐test, the presence of drugs with sedative properties will result in a decrease in locomotion, which is indicative of a reduced interest in exploring new environments (Moniruzzaman et al. 2015). Locomotor activity serves as a measure of mental alertness, with decreased locomotion indicating tranquility and drowsiness, which may be interpreted as a reduction in central nervous system excitability (Islam et al. 2015). In the event of the soluble fractions of MES, PES, TES, CES, and AES had a statistically significant sedative effect (*p* < 0.05) in comparison with the other test substances. Significant statistical findings were seen for the PES 400 mg/kg dosage at 30 min and the CES 400 mg/kg dose at 90 min after the extract was administered, with a *p*‐value of less than 0.001.

Both humans and animals can induce sleep with thiopental sodium, a barbiturate. The study used the thiopental sodium‐induced sleeping duration test in mice to examine the effects of sedative‐hypnotic drugs (Moniruzzaman et al. 2015). Our investigation found that when *E. sessile* extract TES, CES, and AES were orally administered at a dosage of 400 mg/kg, there were highly statistically significant outcomes (*p* < 0.001) in both the start and duration of sleep. These findings were identical to those seen with diazepam. There is substantial data indicating that CNS depressive barbiturates, such as thiopental sodium, bind to the barbiturate binding site on the GABA receptor complex and promote GABA‐mediated hyperpolarization of postsynaptic neurons (Schug et al. 2018). Our findings suggest a strong correlation between the sedative properties of *E. sesssile* and diazepam.

Based on the molecular docking studies, it appears that this plant has analgesic properties, as all of the chemicals tested docked favorably with cyclooxygenase‐1 (PDB ID: 5wbe) and human cyclooxygenase‐2 (PDB ID: 5ikq). In relation to COX‐2, COX‐1 has exhibited a notable affinity for the chemical due to its analgesic characteristics. Ten compounds were selected to bind to the COX‐1 and COX‐2 receptors on the basis of their docking scores. Every chemical we investigated in our molecular docking study interacted with all of the target proteins. As a result of its binding energies of −8 kcal/mol to Stigmasta‐5,22‐dien‐3‐ol, acetate, (3‐beta), and −7.8 kcal/mol to retinoic acid, the COX‐1 enzyme (PDB ID: 5wbe) had the most binding power. Additionally, a number of compounds highly affinized the COX‐1 receptor. Cyclooxygenase‐2 enzyme is primarily responsible for acute pain, and specific COX‐2 inhibitors are a superior alternative to block the pain‐stimulating effect. However, COX‐2 inhibitors have been linked to various adverse effects, necessitating the use of selective COX‐2 inhibitors or COXIB medicines to specifically antagonize the COX‐2 enzyme (Aziz et al. 2019). All the substances significantly enhanced their analgesic effects by blocking the COX‐2 receptor (PDB ID: 5ikq). The compounds Stigmasta‐5,22 dien‐3‐ol acetate and retinoic acid had a strong binding affinity of −7.4 cal/mol.

These results show that our chemicals can stop the production of prostaglandins by going after the active site residues of Cyclooxygenase‐1 and Cyclooxygenase‐2.

## 5 Conclusion

The most recent study found that different extracts of *Elatostema sessile* contained various phytochemicals that were antioxidant, cytotoxic, strong pain killing, and CNS depressant. This botanical specimen may have made a substantial contribution to the field of herbal medicine research. Nevertheless, more investigation is required to reveal the precise physiological impacts of the subject. The computational analysis revealed that the chemicals found using GC–MS had significant affinities for COX‐1 and COX‐2, indicating possible pathways for pain relief in *E. sessile* . The drug‐like characteristics of the discovered components were assessed using ADME/T analysis after GC–MS analysis. Further investigation is required to isolate and characterize the bioactive constituents of *E. sessile* to have a comprehensive understanding of its biological properties.

## Author Contributions

**Aninda Kumar Nath:** conceptualization (lead), formal analysis (equal), supervision (lead). **Saima Sultana Alam:** investigation (supporting), methodology (equal), software (equal). **Joushan Ara:** investigation (equal), methodology (equal), visualization (equal), writing – original draft (equal), writing – review and editing (equal). **Md. Mustafiz Chowdhury:** data curation (lead), formal analysis (lead), investigation (equal), methodology (lead), resources (equal), software (equal), writing – original draft (lead), writing – review and editing (lead). **Ummah Tasnim Nisat:** methodology (equal), resources (equal), software (equal), visualization (equal). **Mohammad Jamal Uddin:** data curation (equal), software (equal), visualization (equal), writing – review and editing (equal). **Md. Tanvir Chowdhury:** data curation (equal), software (equal), validation (equal). **Sabbir Khan:** resources (equal), software (equal), visualization (equal). **Mycal Dutta:** conceptualization (equal), data curation (equal), formal analysis (equal), project administration (equal), resources (equal), supervision (equal), validation (equal), visualization (equal).

## Consent

The authors have nothing to report.

## Conflicts of Interest

The authors declare no conflicts of interest.

## Data Availability

The datasets used and/or analyzed during the present study are available upon reasonable request from the relevant author, and they were also discovered online by searching Google Scholar, PubMed, websites, book chapters, etc.
